# Human Articular Chondrocytes Regulate Immune Response by Affecting Directly T Cell Proliferation and Indirectly Inhibiting Monocyte Differentiation to Professional Antigen-Presenting Cells

**DOI:** 10.3389/fimmu.2016.00415

**Published:** 2016-10-24

**Authors:** Rui C. Pereira, Daniela Martinelli, Ranieri Cancedda, Chiara Gentili, Alessandro Poggi

**Affiliations:** ^1^Regenerative Medicine Unit, Department of Experimental Medicine, University of Genova, Genova, Italy; ^2^Molecular Oncology and Angiogenesis Unit, Department of Integrated Oncological Therapies, IRCCS AOU San Martino IST, Genova, Italy

**Keywords:** allogenic cells, T lymphocyte, immune response, antigen-presenting cells, dendritic cells, chondrocyte implantation

## Abstract

Autologous chondrocyte implantation is the current gold standard cell therapy for cartilage lesions. However, in some instances, the heavily compromised health of the patient can either impair or limit the recovery of the autologous chondrocytes and a satisfactory outcome of the implant. Allogeneic human articular chondrocytes (hAC) could be a good alternative, but the possible immunological incompatibility between recipient and hAC donor should be considered. Herein, we report that allogeneic hAC inhibited T lymphocyte response to antigen-dependent and -independent proliferative stimuli. This effect was maximal when T cells and hAC were in contact and it was not relieved by the addition of exogenous lymphocyte growth factor interleukin (IL)-2. More important, hAC impaired the differentiation of peripheral blood monocytes induced with granulocyte monocyte colony-stimulating factor and IL-4 (Mo) to professional antigen-presenting cells, such as dendritic cells (DC). Indeed, a marked inhibition of the onset of the CD1a expression and an ineffective downregulation of CD14 antigens was observed in Mo–hAC co-cultures. Furthermore, compared to immature or mature DC, Mo from Mo–hAC co-cultures did not trigger an efficacious allo-response. The prostaglandin (PG) E_2_ present in the Mo–hAC co-culture conditioned media is a putative candidate of the hAC-mediated inhibition of Mo maturation. Altogether, these findings indicate that allogeneic hAC inhibit, rather than trigger, immune response and strongly suggest that an efficient chondrocyte implantation could be possible also in an allogeneic setting.

## Introduction

Articular cartilage, the joints load-bearing tissue, has limited repair and regeneration capacity (1). Chondrocytes, the only cell type present in articular cartilage comprising only 2–5% of the total cartilage volume, are responsible for regulating the integrity and homeostasis of the tissue by their ability to synthesize the structural components of the extracellular matrix (ECM) (2). Cartilage lesions caused by trauma or diseases have been recognized as a cause of significant morbidity, affecting a significant part of the worldwide population. As consequence, after uncontrolled inflammation courses, the unique therapeutic option to restore or replace the dysfunctional tissue is the transplantation of somatic chondrocytes or stem cells with or without the use of guided biomaterials (3–5). Successful outcome of cell-based cartilage engineering depends on cells ability to form homeostatic and functional tissue, with the appropriate formation of cartilaginous matrix to achieve the load-bearing capabilities of the natural tissue. Autologous chondrocyte implantation (ACI) is the current gold standard cell therapy for cartilage lesions (6). ACI, like others autologous therapies, requires articular tissue to be removed from healthy area of the knee cartilage, which rises donor site morbidity associated with reduction of function and increasing of pain over time (7). With the use of autologous cells in ACI, the immunological barriers to transplantation are fairly low. On the other hand, an allogeneic chondrocyte therapy would cancel the donor site morbidity and reduce time and costs while retaining the regenerative component of the cellular therapy. In this case, the successful transplantation of allogeneic cells requires the overcome of several immunological barriers involved in allo-recognition phenomena during an inflammatory response process. Also, it has been reported that although human articular chondrocytes (hAC) can express HLA-DR and ICAM1, they do not trigger an efficient allogeneic immune response (8). Furthermore, hAC can substitute monocytes in triggering T cell proliferation to mitogenic polyclonal stimuli, such as phytohemoagglutinin A (PHA) (8). Altogether these findings would support the idea that hAC can actively interact with lymphocytes and influence their proliferative response. Thus, it is relevant to define whether human chondrocytes may affect T cell response specifically triggered through CD3 T cell receptor complex and why T lymphocytes cannot recognize allo-chondrocytes. It is well established that T cells can respond to foreign peptide antigen associated with major hystocompatibility molecules expressed on professional antigen-presenting cells (APC) as dendritic cells (DC) (9). In this work, we have analyzed the ability of allogeneic hAC to regulate T cell-mediated immune response and the effect exerted on the differentiation of peripheral blood (PB) Mo to DC. Indeed, the use of allogeneic chondrocytes in cartilage defects and osteoarthritis (OA) disease, characterized by articular cartilage loss and synovial inflammation (10), should be further improved with the knowledge of the kind of interaction between hAC and immune cells.

## Materials and Methods

### Monoclonal Antibodies and Reagents

Monoclonal antibodies (mAbs) specific for CD3 (UCHT-1, IgG1 a gift of PCL Beverley, London and JT3A, IgG2a), anti-HLA-I (3A3, IgM, a gift of Dr E. Ciccone, University of Genoa), anti-HLA-DR (D1.12, IgG2a, a gift of Dr. R. Accolla, University of Insubria, Varese), anti-CD33 (ATCC, IgG1, HB-10306), anti-CD34 (ATCC, HB-12346, IgG1), anti-CD44 (T61/7, IgG1), anti-CD45 (ATCC, 9.4, IgG2a), anti-CD73 (ATCC, IgG2b, HB-10744), anti-CD105 (ATCC HB-10743, IgG1), CD31 (89D3, IgG2a), anti-CD25 (9.3, IgG1) anti-CD16 (KD1, IgG2a), anti-CD14 (63D3, IgG1, a gift of Dr D. Vercelli, HSR San Raffaele, Milan) were used as culture supernatant. Anti-CD1a-FITC, anti-CD80-Vio, anti-CD83-APC, anti-CD86-PE, and anti-CD14PE-Cy7 were from ExBioPraha, Goat anti-mouse anti-isotype-specific second reagent conjugated with PE or Alexafluor 647 and beads coated with anti-CD3 and anti-CD28 mAbs were from Life Technologies (Milan Italy) and used according to instructions of manufacture. TGFβ-1, granulocyte monocyte colony-stimulating factor (GM-CSF), and IL-4 were from Peprotech Inc. (London, UK). Recombinant human interleukin (IL)-2 was from Miltenyi Biotech. Adenosine, kynurenine, prostaglandin E_2_ (PGE_2_), bacterial lyposaccharide (LPS), PHA, and staphyloenterotoxin B (SEB) fetal bovine serum (FBS) for lymphocytes, hyaluronidase, collagenase I, collagenase II, trypsin, dexamethasone, ascorbic acid, and phosphate-buffered saline (PBS) were from Sigma Chemical. All chemicals and bacterial-derived substances were used in a biosafety level 2 laboratories according to the safety procedures of IRCCS AOU San Martino IST. Coon’s modified HAM’s F-12 medium was from Biochrom AG (Berlin Germany), glutamine, FBS for hAC and Ficoll Hypaque for separation of lymphocytes were from Euroclone (Milan, Italy). RPMI1640 medium for lymphocyte cultures was from GIBCO and (CFSE) (Life Tecnologies, Milan, Italy). ATPLite assay kit was from Perkin Elmer (Monza, Italy). The negative selection kits for isolation of T cells and CD4^+^ T cells or do-it-yourself kit for positive selection of CD14^+^ monocytes were purchased from Stemcell Tecnologies (Vancouver, Canada).

### Specimens of Human Articular Chondrocytes and Peripheral Blood Mononuclear Cells

Human articular chondrocytes were harvested from adult articular cartilage from the femoral condyles of 20 patients undergoing partial knee replacement (age mean = 70 years, *n* = 20). Peripheral blood mononuclear cells (PBMC) were obtained from buffy coats (*n* = 35, age mean 45 years, range 18–50 years) of healthy donors and used within 1-day gathering. All biological samples were obtained in consensus with the guidelines and with the approval of the Institutional Ethics Committee and after signed institutional informed consent.

### Isolation and Culture of hAC

Human articular chondrocyte were obtained as described by Pereira et al. (11). In brief, single articular chondrocytes were released by repeated digestions using an enzymatic solution composed by 1 mg/ml hyaluronidase, 400 U/ml collagenase I, 1000 U/ml collagenase II, and 0.25% trypsin in PBS. Isolated cells were united and cultured in 2D adhesion using Coon’s modified Ham’s F-12 supplemented with 10% FCS, 2 mM glutamine, 100 U/ml penicillin, and 100 μg/ml streptomycin. At ~90% of confluence, cells were detached and plated at different densities. During culture, cells were monitored using a bright field microscope (Axiovert 40 CFL) equipped with a digital camera (Canon Power Shot G8).

### Chondrogenic Differentiation Assay

Chondrogenic potential of hAC was investigated by the pellet culture (12). About 2.5 × 10^5^ cells were pelleted in 15 ml conical tubes and cultured for 21 days in the chondrogenic medium; Coon’s F-12 modified Ham’s medium supplemented with human transforming growth factor-β1 (10 ng/ml), dexamethasone (10^−7^ M) and ascorbic acid (50 mg/ml) according to Johnstone et al (13). Media were changed every 2 days. Tissue characteristics were evaluated by histology. Moreover, we test ectopic cartilage formation in athymic mice; 3D pellets were maintained for 2–3 days *in vitro* before subcutaneous implantation in CD-1 nu/nu mice (Charles River Italia). Animals were sacrificed, and implants were recovered after 4 weeks for the histological analysis of cartilage formation (14). All animals were maintained in accordance with standards of the Federation of Eu-Laboratory Animal Science Association, as required by the Italian Ministry of Health and with the approval of the Institutional Ethic Committee (Research project n.336).

### Histology and Immunohistochemistry Characterization

Pellets and recovered implants were fixed in 4% formaldehyde in PBS, dehydrated in ethanol, and paraffin embedded. Cross sections (5 μm) were cut, dewaxed, and stained with toluidine blue for detection of sulfated glycosaminoglycan. For immunohistochemical analysis, sections were dewaxed and treated with methanol:hydrogen peroxide (49:1) for 30 min and then treated with 1 mg/ml hyaluronidase in PBS (pH 6.0) for 30 min at 37°C and washed with PBS. Slices were then incubated with goat serum for 1 h to reduce non-specific binding. The type II collagen antibody diluted 1:250 (CIICI anti-COLLII, DSHB, University of Iowa) was added and incubated for 1 h at room temperature. The procedure was performed using a Histomouse Kit (Zymed Laboratories). Detection was detected with the biotinylated secondary antibody and streptavidin–peroxidase. The oxidase activity was visualized by the AEC (3-amino-9-ethylcarbazole) chromogen substrate. Histology and immunohistochemistry slides were observed at different magnifications and images acquired with the Axiovert 200M microscope (Carl Zeiss).

### Gene Expression Characterization

Total RNA was extracted from hAC using Trizol^®^ reagent according to the manufacture’s instructions (Invitrogen, CA, USA) and kept at −80°C for subsequent RNA extraction (14). Briefly, cells were incubated at 4°C for 10 min with chloroform (Sigma) and centrifuged at 13000 rpm for 15 min; 700 μl supernatant were collected and an equivalent volume of isopropanol (Sigma/I-9516) was added. After RNA precipitation, samples were centrifuged at 13000 rpm and 4°C for 15 min. The supernatant was removed and 700 μl of 70% ethanol was added. Tubes were again centrifuged at 13000 rpm at 4°C for 5 min, and the supernatant was removed. The pellets were left to air-dry at RT and at the end were resuspended in 50 μl DNase/RNase-free distilled water (Gibco/10977-015). RNA content and integrity was assessed using a NanoDrop (NanoDrop Technologies, USA). Isolated RNA was transcribed into cDNA using the iScript cDNA synthesis kit (1708891). Gene expression levels were quantified by real-time quantitative RT-PCR (qPCR) using ABI Prism 7700 Sequence Detector (Applied Biosystems) according to the manufacturer’s instructions and using the primers reported in Table 1. Data were analyzed for the gene of interest and normalized for the housekeeping gene glyceraldehyde-3-phosphate dehydrogenase (GAPDH) using ΔCT expression ratio following MIQE guidelines.

**Table 1:** **Primers used to evaluate the gene expression of human articular chondrocytes by real-time quantitative PCR**.

Gene	Forward	Reverse
GAPDH	5′-ACAGTCAGCCGCATCTTCTT-3′	5′-ACGACCCAAATCCGTTGACTC-3′
Collagen type I	5′-CATCTCCCCTTCGTTTTTGA-3	5′-CCAAATCCGATGTTTCTGCT-3′
Collagen type II	5′-GACAATCTGGCTCCCAAC-3′	5′-ACAGTCTTGCCCCACTTAC-3
Aggrecan	5′-TGAGTCCtTCAAGCCTCCTGT-3′	5′-TGGTCTGCAGCAGTTGATTC-3′
SOX-9	5′-TACGACTACACCGACCACCA-3′	5′-TTAGGATCATCTCGGCCATC-3′

### Isolation of PBMC, PBMC–hAC Co-Cultures Experiments, and Evaluation of T Lymphocyte Proliferation

Peripheral blood mononuclear cells were separated from blood samples of healthy donors as described (15). hAC the day before the co-culture experiment were detached from culture flask, washed and 10^5^, 0.5 × 10^5^ and 0.25 × 10^5^ cells were seeded in 96 flat-bottomed microwells plates in RPMI 1640 medium supplemented with10% of FBS. The day after the culture medium was discarded and hAC were co-cultured with PBMC at different PBMC:hAC ratios (20:1, 10:1, 5:1) in RPMI 1640 supplemented with 10%FBS, glutamine, and antibiotics or in the presence of anti-CD3mAb (JT3A. IgG2a, 20 ng/ml), PHA (5 μg/ml), or SEB (10 ng/ml) in U-bottomed microplates in 200 μl and proliferation kinetics was evaluated at 3, 5, and 7 days of culture. Cell proliferation was assessed after labeling PBMC with CFSE according to manufacturer’s instructions. Proliferation was assessed by cytofluorimetric evaluation of progressive loss of CFSE proportional to cell division, on gated T cells labeled with anti-CD3 mAb (UCHT-1, IgG1) followed by AlexaFluor 647-conjugated anti-isotype specific goat anti-mouse antiserum (Life Technologies, Milan, Italy) (16). In preliminary experiments, this assay gave comparable results to assays based on uptake of radiolabeled thymidine or evaluation of ATP content by ATPlite assay kit (Perkin Elmer, Waltham, MA, USA). In some experiments, recombinant IL-2 (2 ng/ml, Miltenyi Biotech, Germany) was added on day 2 to determine at days 3,5, and 7 the responsiveness of lymphocytes to this cytokine and determine the kinetics of response to IL-2. To determine the role of hAC–PBMC contact in regulating lymphocyte cell proliferation, in some experiments, PBMC were seeded on Millicell transwell^®^ (TW) with 0.3 μm pores (Millipore Corporation, Billerica, MA, USA) put in 24-well plates with hAC seeded on the bottom to avoid hAC–PBMC contact. In some experiments, hAC and PBMC were co-cultured in 24-well plates and CFSE labeled PBMC put in TW in the same culture well. The ratio between PBMC and hAC was the same as when cultured in flat-bottomed microplates; indeed, the number of cells in culture was proportional to the plastic growth area. In this setting, soluble factors produced during the PBMC–hAC contact in the lower chamber can diffuse in the upper TW and their influence on response to proliferating signals can be detected. Isolation of CD4^+^ T cells was performed using Rosettesep negative isolation kit for CD4^+^ T cells following manufacture’s instructions (Stemcell Technologies, Vancouver, BC, Canada). The purity of CD4^+^ T cell populations was always >95% (ranging from 95 to 100%). CD4^+^ T cells were co-cultured with hAC at the CD4^+^: hAC ratio of 5:1 and proliferation to CD3–CD28 mAb-coated beads evaluated on days 3, 5, and 7. The same samples were also analyzed for the expression of CD25 (IL-2Rα) with anti-CD25 mAb (clone 93, IgG1) followed by Alexa 647-conjugated goat anti-mouse on days 2, 4, and 6 to analyze the kinetics of expression. In some experiments, 2 ng/ml of IL-2 was added on day 2 of the co-culture experiments.

### Isolation of Peripheral Blood Mo, Generation *In Vitro* of Dendritic Cells, and Co-Cultures with hAC

CD14^+^ Mo were positively isolated from PBMC with Easy-sep “do-it-yourself kit” (Stemcell Technologies) using the anti-CD14 mAb clone 63D3 IgG1 isotype (Biolegend, San Diego, CA, USA). The purity of CD14^+^ cells was more than 85% (range from 85 to 95%). To generate immature (i) DC, CD14^+^ Mo were cultured with 20 ng/ml of GM-CSF and 20 ng/ml of IL-4 for 6 days as previously described (9, 17). Additional culture of immature DC (iDC) with LPS (1 μg/ml) induces their differentiation to mature (m) DC (9, 17). Generation of iDC was characterized by the downregulation of CD14 antigen and neo-expression of CD1a. Maturation of iDC to mature DC (mDC) was evidenced by the upregulation of accessory molecules as CD80, CD83, and CD86, co-culture experiments with hAC were performed in 24-well microplates (Euroclone) at different Mo:hAC ratios of 10:1, 20:1, 40:1, 80:1, 160:1. The co-cultures experiments were performed in contact or seeding Mo in Millicell transwell (Millipore, TW) as indicated for co-cultures with PBMC. Conditioned media (CM) from the different conditions were collected, centrifuged at 10,000 rpm for 10 min, and stored at −80C for further analysis.

### Prostaglandin E_2_ (PGE_2_) Quantification by ELISA

Determination of PGE_2_ content in conditioned media (CM) from culture of Mo and hAC with GM-CSF and IL-4 differentiation cytokines was performed using a PGE_2_ specific ELISA kit (Cayman, Ann Arbor, MI 48108, USA). Content of PGE_2_ in CM of control cultures of Mo with GM-CSF and IL-4 as well as that from hAC was analyzed for comparison. The CM was harvested on day 4 of culture to allow production of soluble factors but avoiding their consuming as defined in preliminary experiments. Results are expressed as picogram/milliliter of PGE_2_.

### Cytofluorimetric Assays

In indirect immunofluorescence assay cells were stained with the indicated mAbs followed by incubation with anti-isotype specific goat anti-mouse antiserum conjugated with the fluorochromes indicated. Samples were run on a CyAN ADP cytofluorimeter equipped with three lasers (Vio405, Argon 488, and He-Neon 613), compensation between the different fluorochromes was performed by analyzing samples stained with single antibodies and by auto-compensation analysis. At least 10^5^ events were analyzed in each instance and data are represented as Log fluorescence intensity vs. number of cells (single immunostaining) or as Log fluorescence intensity vs. Log fluorescence intensity in double or multicolor (5-color immunostaining for Mo differentiation) immunofluorescence. In some experiments, also physical parameters as forward scatter (FSC) and side scatter (SSC) were shown to indicate the cell populations analyzed.

Cell populations derived from co-cultures of Mo and hAC did not contain detectable number of the last ones. This notion was supported by the findings that the amount of large cells characterized by high FSC and SSC harvested from Mo cultured with hAC was negligible (see Figure S2C in Supplementary Material). In addition, the initial number of hAC put in culture with Mo was of about 5 × 10^4^ at a 10:1 Mo:hAC ratio (that means 5 × 10^5^ Mo) after 6 days of cultures only 10 × 10^4^ of floating cells were put in culture with allo-T lymphocytes. Thus, as about 1–3% of cells did not express markers of Mo–DC, the putative amount of hAC could be about 1–3% of 10 × 10^4^ cells (10–30 × 10^2^ of total cells). The ratio in allo-test was 10:1 T vs. DC that is about 100–300 of total hAC. This amount is really too low to get T cell inhibition evident at 10:1 and 5:1 PBMC:hAC ratios. In addition, virtually all cells harvested from co-cultures with hAC were CD45^+^ (a marker absent on hAC), excluding the possibility that residual hAC were present during allo-response assay.

### Mixed Lymphocyte Reaction Using iDC, mDC, and Mo Cultured with hAC, hAC as Stimulator in MLR and as Third Party in MLR Assay

Mixed lymphocyte alloreaction (MLR) was performed with T cells isolated from PBMC by negative Rosettesep kit (Stemcell Tecnologies, Vancouver, BC, Canada) following manufacture’s instructions modified (15). The purity of T cell populations obtained was always more than 98% (range 98–100%). T cells were put in culture in 200 μl/well in flat-bottomed plates in the presence of allogeneic iDC, mDC, or Mo cultured with hAC for 6 days as described in Section “Chondrogenic Differentiation Assay.” These APC were harvested from cultures and washed three times and seeded at 10:1 T:APC ratio (10^5^ T cells vs. 10^4^ APC). The purity of these APC was always >99% as assessed by the expression of APC specific markers CD86 and CD45 leukocyte common antigen (not shown). Proliferation was evaluated by ATPLite assay kit (Perkin Elmer) on day 6 of culture. Proliferation of only T cells (negative control), or T cell and Mo (as a measure of MLR in the presence of unprofessional APC) or T cells in the presence of CD3–CD28-coated beads (as maximal level proliferation of T cells) was analyzed for comparison. In this case, proliferation was assessed as ATP content of cell cultures as the number of iDC, mDC, Mo, and Mo derived from co-cultures with hAC were limited and this assay was adequate to perform several replicates of the same culture condition and so many different combinations. Indeed, ATPlite assay gave similar results (evaluated as degree of proliferation) of those obtained with CFSE labeling, in preliminary experiments. This allowed us to use ATPlite instead of CFSE labeling in some selected experiments. In other experiments, hAC have been used as stimulator in co-cultures with allogeneic PBMC at the PBMC:hAC ratio of 10:1 (10^5^ PBMC vs. 10^4^ hAC) in flat-bottomed 96-well microplates. In these experiments, hAC were seeded 2 days before the allogeneic PBMC. PBMC was labeled with CFSE and proliferation analyzed as decrement of CFSE content in CD3^+^ gated T cells. Some MLR assays were performed on hAC as third party at the responder PBMC:hAC ratio of 10:1 (10^5^ PBMC vs. 10^4^ hAC); in these experiments, stimulator was represented by another irradiated PBMC. In this case, proliferation of responder T cells was evaluated as decrement of CFSE content of CFSE labeled responder PBMC.

### Statistical Analysis

Statistical analysis of results was performed applying the two tails student *t*-test on at least *n* = 6 independent experiments using different donors of hAC and PBMC or APC. Data were considered statistically significant with a *p* < 0.05 and *p*-value is indicated with an asterisk (*).

## Results

### Cultured hAC Maintain Cartilage Somatic Functional Properties *In Vitro*

**Figure 1:**
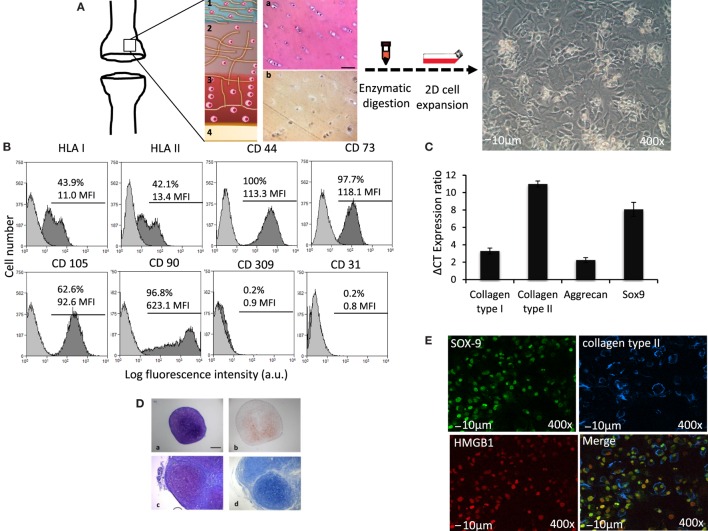
**Phenotypic features of hAC**. **(A)** Schematic representation of surgical specimens of articular cartilage (on the left), enzymatic digestion and 2D cell expansion in culture (middle) and images of cultured hAC (on the right). 1, 2, 3, and 4 represent schematically the anatomic architecture of cartilage divided by zones: (1) superficial tangential; (2) middle; (3) deep, and (4) calcified cartilage zone. Histological explants characterization: (a) hematoxylin and eosin and (b) immune detection of collagen type II. **(B)** Phenotype of hAC assessed by cytofluorimetry with mAbs to the indicated surface molecules (HLA-class I, HLA-class II DR antigens, CD44, CD73, CD105, CD90, CD309, and CD31). Results are shown as log fluorescence intensity vs. number of cells. In each subpanel are shown the negative control (light gray histogram) and the expression of the indicated antigens (dark gray histogram). In each panel are indicated the % of positive cells and their mean fluorescence intensity (MFI) in arbitrary units (a.u.). **(C)** ΔCT ratio of mRNA expression in cultured hAC of either collagen type I or type II, or aggrecan or sox-9 transcription factor and GAPDH housekeeping gene. **(D)** Histological staining of 3D pellet culture cross sections. (a,b) *In vitro* pellet formed by human articular chondrocytes. (a) toluidine blue staining, (b) immunohistochemical expression of collagen type II. (c,d) *In vivo* subcutaneous implantation of pellets. Pellets were formed by hAC, after 2 weeks in culture, these pellets were then implanted in nude mice. (c) toluidine blue staining, (d) alcian staining. Scale bar = 100 μm. **(E)** Expression of nuclear Sox9 (green) and extracellular collagen II (blue) on hAC assessed by confocal microscopy. The expression of HMGB1 (red) as a nuclear marker is shown to identify nucleus. The merge image is also shown. Bar = 10 μm. 400× magnification.

*Ex vivo* isolation and culture of adult hAC gives raise to cells with restrict maintenance of their somatic assets. One of the main limitations of using these adult somatic cells in tissue regeneration therapies is the limited capacity to preserve their phenotype and genotype memoir over time (18). Figure 1 illustrates how isolation of hAC was performed and the morphological features of hAC *in vitro* cultured (Figure 1A). Cytofluorimetric analysis (Figure 1B) indicated that hAC can express, although at low level, HLA class I and II antigens; indeed, the mean fluorescence intensity (MFI) of HLA-I expressed on leukocytes was 100 times higher than MFI that showed by hAC; furthermore, on PB Mo HLA-DR was about 500 times more expressed than on hAC (not shown). Most interestingly, the expression of both HLA antigens on hAC appeared to be bimodal. On the other hand, CD44, CD73, CD105, and CD90 markers were present on hAC but not CD309, CD31 (Figure 1B) CD45, CD14, CD34, CD33, CD80, and CD86 (not shown). It is of note that CD44, CD73, and CD105 showed a monomodal and quite homogeneous expression. The maintenance of the cartilage phenotype of the cultured hAC was further confirmed by the mRNA expression of typical markers of hAC, such as collagen type II, aggrecan, and sox-9 transcription factor (Figure 1C). Furthermore, the cultured cells retained the ability to form cartilage both in a 3D differentiation system *in vitro* and when implanted *in vivo* (Figure 1D). Finally, hAC did show a clear reactivity with sox9 and collagen type II by indirect immunofluorescence and confocal microscope analysis (Figure 1E).

Altogether, these findings were consistent in all the primary cultures analyzed (*n* = 20), and hAC were used in functional assay independently from the age of the original specimen from which they were isolated. Indeed, hAC maintained their differentiation potential throughout the culture period of 2 months as previously described (14).

### Peripheral Blood T Lymphocyte Proliferation Is Regulated by Cultured hAC

To define whether hAC can regulate T lymphocyte proliferation to mitogenic stimuli, we labeled PBMC with CFSE, put them in culture with hAC in the presence of anti-CD3 mAbs, PHA, or SEB, and decrement of CFSE content in association with identification of T lymphocytes by CD3 antigen expression was analyzed by flow cytometry. The use of these three different mitogenic stimuli was performed to clarify whether the effect of hAC was dependent on the kind of stimulus employed to trigger proliferation. Indeed, PHA can trigger T cells through the interaction with several activating receptors expressed on T cells while SEB can preferentially trigger T lymphocyte through the interaction with β-chain of T cell antigen receptor (TCR) (19). Anti-CD3 mAb can mimic the triggering elicited through TCR as it is well established that CD3 is the molecular complex associated with TCR, which delivers the signal inside the lymphocyte upon the specific antigen interaction with TCR (19–21). Furthermore, PHA can bind several sugar moieties expressed on molecules that can be also expressed on non-T non-B lymphocytes as Mo and natural killer cells. These cells can have a role in supporting T lymphocyte proliferation (19, 22). Thus, these three types of stimuli can mimic different physiological conditions upon which T cells can respond. Most importantly, all the stimuli used are effective when in the cell culture are present functional monocytes that can help T cell to proliferate giving to T cells the second accessory signal needed for an efficient immune response (9).

**Figure 2:**
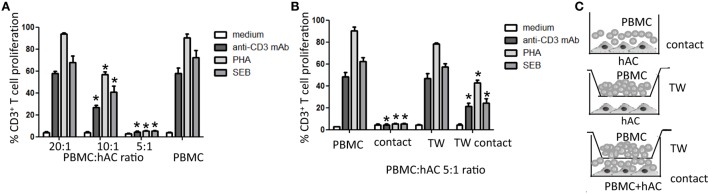
**Human articular condrocyte (hAC) regulate T cell proliferation**. **(A)** T cell proliferation of CD3^+^ T cells with the indicated stimuli at different PBMC:hAC ratio (20:1, 10:1, 5:1) or PBMC alone (medium). **(B)** T cell proliferation of PBMC on hAC at the PBMC:hAC ratio of 5:1 in contact or separated by a Millicell transwell chamber (TW, PBMC in TW) or by PBMC in TW separated by a co-cultures of PBMC and hAC (TW-contact). Results are the mean ± SD of eight different experiments. **(C)** Schematic representation of the different culture conditions: contact **(A,B)**, TW **(B)**, and TW-Contact **(B)**. *p < 0.001 vs. PBMC.

We found that hAC can markedly inhibit T lymphocyte proliferation to any of the stimuli applied (Figures 2A,B; Figure S1 in Supplementary Material). It is to point out that the hAC-mediated inhibiting effect was dependent on the ratio between PBMC and hAC. Indeed, proliferation was abrogated when PBMC:hAC ratio was 5:1 while the inhibitory effect almost disappeared at the PBMC:hAC ratio of 20:1 (Figure 2A). More importantly, we noted that the observed inhibition at PBMC:hAC ratio of 5:1 was evident only when PBMC and hAC were able to interact each other. Indeed, hAC did not exert any inhibiting effect on T cell proliferation when PBMC and hAC were cultured in the same culture well but separated by an insert cell chamber (Figure 2B). This *in vitro* system allowed the diffusion of soluble factors but it did inhibit the PBMC–hAC contact. Furthermore, we found that soluble factors produced upon PBMC–hAC interaction can affect lymphocyte proliferation; indeed, PBMC seeded in insert cell chamber displayed less proliferation when in the same culture well in the lower chamber hAC and PBMC were in contact (Figures 2B,C to clarify the different experimental conditions). Altogether, these findings suggest that hAC upon contact with PBMC can deliver an inhibiting signal to T cell proliferation; this signal is also mediated by soluble factors produced during hAC–PBMC interaction.

### hAC Inhibit CD4^+^ T Cell Proliferation Triggered through the Engagement of CD3 and CD28 Surface Receptors

**Figure 3:**
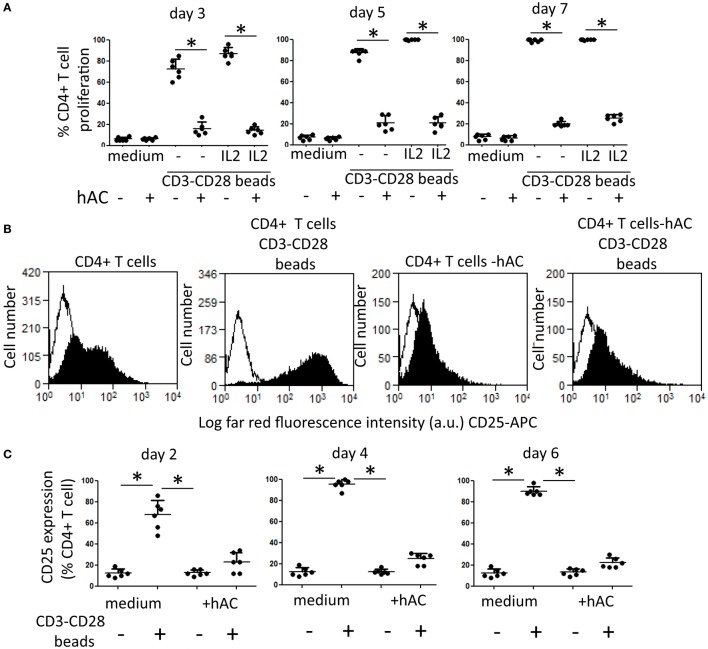
**Effect of hAC on CD4^+^ T cell proliferation and IL-2R expression triggered through the engagement of CD3 and CD28 surface receptors**. **(A)** CD4^+^ T cell proliferation to coated beads with anti-CD3 and anti-CD28 mAb at the time points indicated (3, 5, and 7 days). IL-2 (2 ng/ml) was added on day 2 of culture. Medium: proliferation of CD4^+^ T cells without mAb-coated beads. Results are the mean ± SD of six independent experiments. *p < 0.001 of CD4^+^ T cell-hAC co-cultures vs. CD4^+^ T cells alone. **(B,C)** CD4^+^ T cells stimulated with CD3–CD28 beads were analyzed for the expression of CD25 (IL-2Rα chain) on day 2 **(B,C)** 4 and 6 **(C)** by indirect immunofluorescence using specific anti-CD25 mAb followed by isotype specific Alexafluor 647-conjugated goat anti-mouse and FACS analysis. Medium: expression of CD25 antigen in the absence of stimuli. **(B)** A representative experiments out of the six depicted in **(C)**. Results in **(C)** are the mean ± SD of six experiments performed with different donors of CD4^+^ T cells and four donors of hAC. *p < 0.001.

Furthermore, to analyze whether hAC can affect proliferation of T cells by acting directly on this cell population or indirectly by inhibiting Mo dependent co-stimulation of T lymphocytes, we purified CD4^+^ T cells from PBMC and this lymphocyte subset have been assessed for mitogenic response to beads coated with anti-CD3 and anti-CD28 mAb. Indeed, to stimulate proliferation of highly purified CD4^+^ T cells is necessary to give two different activation signals: the first one is mediated through CD3 and the second one through CD28 accessory molecule; thus, CD4^+^ T cells can proliferate in the absence of accessory cells as Mo (19–21). Proliferation of CD4^+^ T cells was assessed at different time points to analyze proliferation kinetics (Figure 3A). We found that CD4^+^ T cells did not proliferate to anti-CD3–CD28 mAb-coated beads when cultured with hAC (Figure 3A; Figure S2A in Supplementary Material). It is of note that CD4^+^ T cells in CD4-hAC co-cultures with anti-CD3–CD28 mAb-coated beads did not show the typical morphology of large blast cells that, on the other hand, could be detected in the absence of hAC (Figure S2B in Supplementary Material). Furthermore, the addition of exogenous IL-2 to cell cultures did not relieve inhibition of CD4^+^ T cell proliferation exerted by hAC (Figure 3A). Altogether, these findings strongly suggest that hAC can directly inhibit T cell activation independently on accessory cells as Mo. The lack of recovery by the addition of IL-2 would indicate that the hAC-mediated inhibiting effect could affect also the IL-2R expression on CD4^+^ T cells. Indeed, it has been found a strong reduction of CD25 expression on lymphocytes when CD4^+^ T cells were co-cultured with hAC and triggered with anti-CD3–CD28 mAb-coated beads (Figures 3B,C).

### Effect of hAC on Monocyte Differentiation to Dendritic Cells

**Figure 4:**
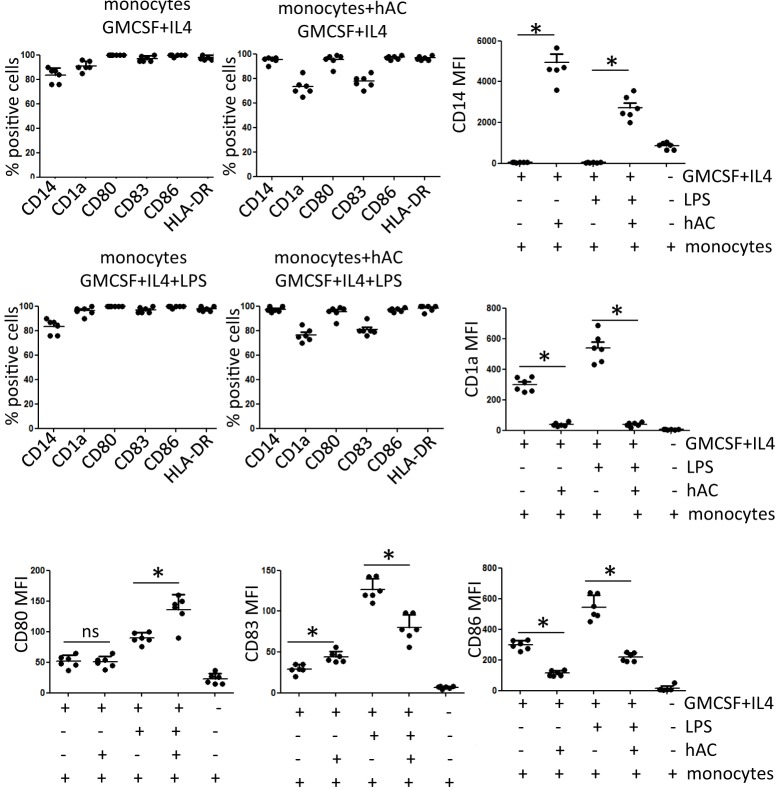
**Expression of differentiation markers of DC in co-cultures with hAC**. The percentage of positive cells as well as the mean of fluorescence intensity of each marker indicated is shown from six independent experiments using four different hAC preparations and six healthy donors of Mo. The expression of CD1a, CD14, CD80, CD83, and CD86 was determined after five-color labeling. The expression of HLA-DR was evaluated by indirect immunofluorescence using anti-HLA-DR specific mAb (D1-12) followed by isotype specific PE-conjugated goat anti-mouse. Results are the mean ± SD of six independent experiments. *p < 0.001.

To deeply analyze the inhibiting effect of hAC on proliferation of T lymphocytes observed using PBMC, we further determined whether hAC could affect differentiation of PB Mo to professional APC as DC. Indeed, it is well established that APC as Mo and DC can play a key role in triggering lymphocytes upon mitogenic stimulation (19–21). Thus, the potent inhibiting effect exerted by hAC on T cell proliferation could be also determined by an impaired differentiation of Mo to DC. To this aim, highly purified Mo (Figure S3 in Supplementary Material) were isolated from PB and cultured for 6 days with GM-CSF and IL-4 to induce their differentiation to the so-called iDC neo-expressing CD1a antigen and upregulating accessory co-stimulatory molecules as CD80, CD83, and CD86. Furthermore, by the addition of LPS and culture for additional 2 days, we analyzed also whether iDC were differentiated to mDC characterized by a stronger expression of CD80 and CD86 accessory molecules [17]. This set of experiments was performed in the presence of hAC at the Mo–hAC ratio of 10 to 1. iDC were harvested on day 6 of culture and analyzed for the surface expression of CD1a, CD14, CD80, CD83, and CD86 (Figure 4 represents results of six independent experiments, Figure S4 in Supplementary Material shows dot plots of a representative experiment). Mo differentiates into iDC upon culture with GM-CSF and IL-4 as they neo-expressed CD1a and the degree of expression of CD14 was strongly reduced. Accessory molecules as CD80, CD83, and CD86 were upregulated in iDC compared to isolated Mo (Figure 4, and in Figure S4 in Supplementary Material compare second row with the first row of dot plots). It is of note that in the presence of hAC–Mo did not differentiate to iDC as CD14 antigen was still expressed at very high levels and CD1a was very slightly upregulated (Figure 4, and in Figure S4 in Supplementary Material compare the third row with the second row of dot plots). Furthermore, the addition of LPS increased the level of expression of CD83 and CD86 on iDC leading to their maturation to mDC (Figure 4); this effect was strongly inhibited in co-cultures with hAC (Figure 4). hAC can affect both the percentage of Mo-derived DC cells expressing a given antigen and the level of its expression indicated as MFI (Figure 4). Although not shown, it should be noted that the inhibition of Mo differentiation to iDC or mDC exerted by hAC appeared to be very strong as it was still maximal at 1:160 hAC:Mo ratio. Altogether, these findings would indicate that hAC could inhibit Mo differentiation to iDC and mDC.

### Mo-Derived Cells Co-Cultured with hAC Did Not Stimulate T Cell Allo-Response

**Figure 5:**
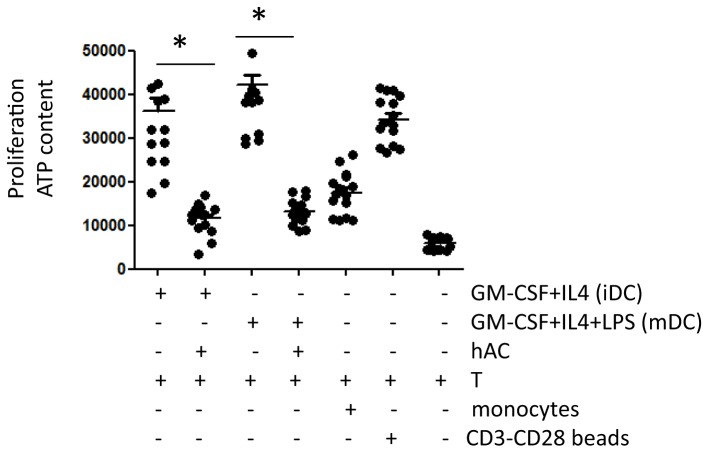
**Mo-derived cells harvested from co-cultures with hAC do not trigger the allo-response of T cells**. Cells derived from culture of peripheral blood Mo with GM-CSF + IL-4 (6 days, indicated as iDC) or GM-CSF + IL-4 + LPS (6 days of GM-CSF + IL-4 + 2 days of LPS, indicated as mDC) without or with hAC were harvested, extensively washed and put again in culture with allo-T cells purified from unrelated donors (T) at the T:antigen-presenting cell ratio of 10:1. The effect of unstimulated Mo [as the minimal triggering of proliferation of T cells and of CD3–CD28 mAb-coated beads (CD3-CD28 coated beads)] as the maximal T cell proliferation is shown for comparison. Results are expressed as counts of luminescence evaluating the ATP content in proliferating cell cultures and they are the mean ± SD of eight independent experiments using six different hAC preparations and eight healthy unrelated T cell donors. *p < 0.001.

To further reinforce the notion that hAC can affect the generation of functional professional DC, we tested whether DC populations from Mo–hAC co-cultures were able to trigger lymphocyte proliferation to allo-antigen (Figure 5). Indeed, iDC and mDC can trigger a stronger response of T cells to allo-antigen than unprofessional APC as Mo (23). Thus, hAC blocking DC generation could impair alloantigen response. To this aim, Mo were cultured with GM-CSF and IL-4 for 6 days either without or with hAC and were further used as stimulator for purified T allo-lymphocytes. This kind of experiment was also performed with cells that were cultured for additional 2 days with LPS, such as mDC. As shown in Figure 5, iDC and mDC can efficiently trigger T cell allo-response; indeed, the level of proliferation of allo-T cells was stronger than that induced by Mo (compare T cell proliferation with iDC, mDC, and Mo as first, third, and fifth group of Figure 5). Importantly, the mitogenic stimulus exerted by iDC or mDC to allo-T cells was comparable to that exerted by anti-CD3 and anti-CD28 coated beads, used as a rate of maximal proliferation of T cells. This indicates that iDC and mDC were indeed professional APC. Moreover, it is of note that Mo-derived populations obtained from co-cultures with hAC did not trigger T cell allo-response. Indeed, proliferation of T cells was slightly higher than that of T cell alone (without any stimulus) and less strong than that triggered by Mo (compare second, fourth group of responses with the fifth of the Figure 5).

### Relevance of Soluble Factors as Mediators of hAC-Dependent Inhibition of Mo Differentiation: Role of PGE_2_

**Figure 6:**
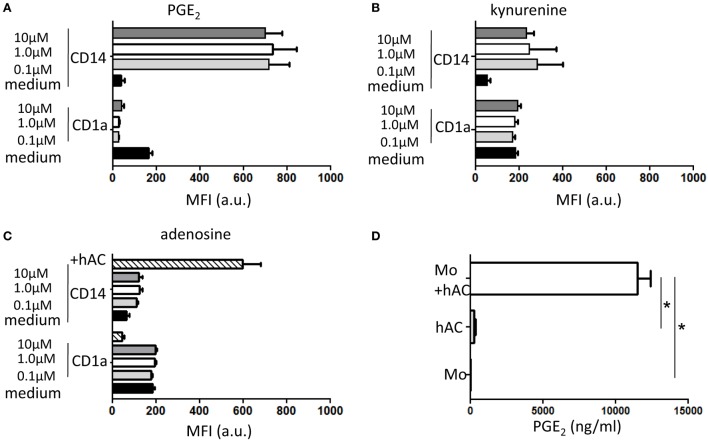
**Effect of PGE_2_, kynurenine, and adenosine on differentiation of Mo to iDC and presence of PGE_2_ in conditioned media of hAC–Mo co-cultures**. To determine the effect on the expression of exogeneous PGE_2_ **(A)** or kynurenine **(B)** or adenosine **(C)**, Mo from PBMC were induced to differentiate to iDC with GM-CSF and IL-4 and incubated starting at the onset of culture with different doses (as indicated) of these three drugs. The expression of CD1a and CD14 antigens were assessed with direct immunofluorescence using CD1a–FITC and CD14PE–Cy7 mAbs on day 6 of culture. Medium indicates the expression of CD1a and CD14 in cultures without the addition of any drug and it represents the typical differentiation of Mo to iDC. In **(C)**, lined bar represents the expression of CD1a and CD14 in co-cultures with hAC showing the impairment in Mo differentiation to iDC. Results are shown as mean of fluorescence intensity of each marker and are the mean ± SD of three independent experiments for each drug. **(D)** The conditioned media (CM) from Mo co-cultured with hAC with GM-CSF and IL-4 was harvested on day 4 of culture and analyzed for the presence of PGE_2_ using a specific ELISA. The amount of PGE_2_ in CM from hAC or Mo is shown for comparison. Results are the mean ± SD of determination of three independent experiments. *p < 0.001.

To define the molecular mechanism underlines the inhibition of Mo differentiation to professional APC, we cultured PB Mo with GM-CSF and IL-4 in the presence of putative soluble inhibitors of Mo differentiations. To this aim, we employed adenosine, kynurenine, and prostaglandin E_2_. Indeed, it has been reported that each of this factor can have a role on Mo differentiation to DC (24); furthermore, kynurenine and PGE_2_ have been reported as relevant soluble mediators secreted in co-cultures with mesenchymal stromal cells (MSC) and Mo able of blocking Mo differentiation to DC. We found that PGE_2_ can inhibit Mo differentiation to DC as it blocked CD1a upregulation and CD14 down regulation characteristically observed in Mo cultured with GM-CSF and IL-4 (Figure 6A). On the other hand, l-kynurenine (10–1–0.1 μM, Figure 6B) and adenosine (10–1–0.1 μM, Figure 6C) did not consistently affect differentiation to DC triggered with GM-CSF and IL-4. These findings prompt us to determine whether conditioned medium (CM) from Mo–hAC co-cultures was able to affect Mo differentiation and contained PGE_2_. As shown in Figure S5 in Supplementary Material, we found that CM from Mo–hAC cultures stimulated with GM-CSF and IL-4 can affect downregulation of CD14 and increase of expression of CD1a. More importantly, PGE_2_ was detectable in this CM (Figure 6D), suggesting that the effect on Mo differentiation exerted by CM could be dependent on the presence of this factor.

### Human AC Do Not Stimulate Allo-Response and Inhibit MLR as a Third Party

**Figure 7:**
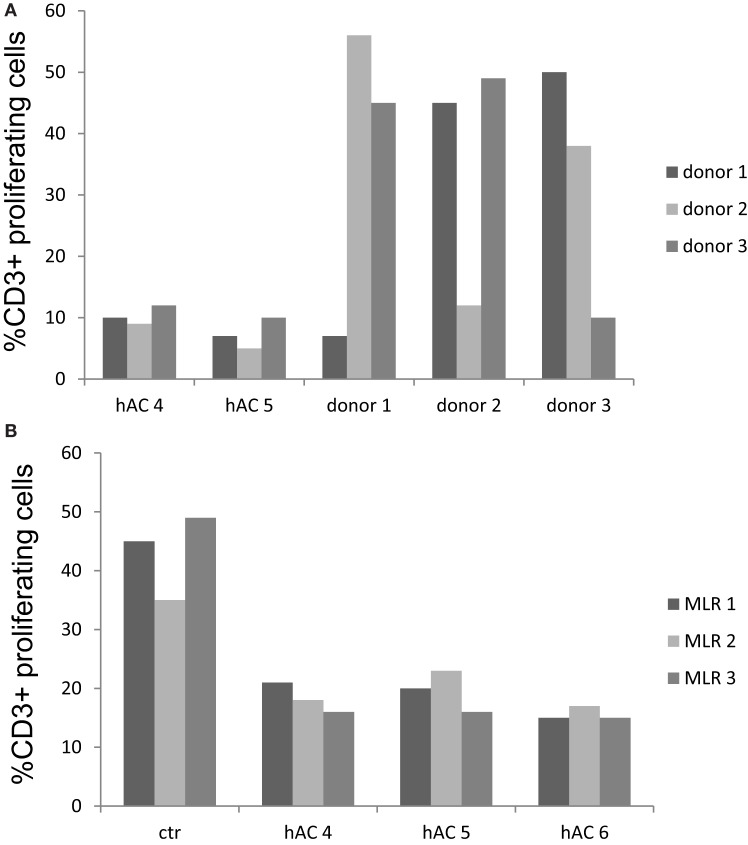
**Human AC do not stimulate allo-response and inhibit MLR as a third party**. **(A)** Allogeneic hAC were used as stimulator of PBMC of three donors labeled with CFSE at the PBMC:hAC ratio of 10:1. Proliferation was evaluated at day 7 and it was assessed as decrement of CFSE content in CD3^+^ T cells (identified by indirect immunofluorescence with anti-CD3 mAb UCHT-1 followed by goat APC-conjugated anti-mouse isotype specific antiserum). Results are expressed as % of CD3^+^ T cells with decrement of CFSE content compared to PBMC cultured alone. MLR between the three different PBMC donors was shown for comparison to define whether PBMC can indeed proliferate upon allo-recognition. **(B)** Regulation of MLR with hAC as a third party. MLR were set up in the presence of hAC (as a third party) at the responder PBMC:hAC ratio of 10:1 and proliferation evaluated as in **(A)** as decrement of CFSE content in CD3^+^ responder T cells. Ctr: level of proliferation of CD3^+^ T cell in the MLR. Results are expressed as % of CD3^+^ T cells with decrement of CFSE content. Responder PBMC cultured alone showed a proliferation of 3–7%.

We further analyzed whether hAC could function as stimulator of allogenic PBMC. For this aim PBMC labeled with CFSE were added to hAC seeded at the PBMC:hAC ratio of 10:1 and proliferation of CD3^+^ T cells was evaluated on day 7 of culture. As shown in Figure 7A, hAC did not trigger proliferation of T cells of three different allogeneic donors, indeed only about 10% of CD3^+^ T cells showed a decrease of CFSE content (Figure 7A, *p* < 0.001, comparing proliferation of leukocytes in co-cultures of hAC and leukocytes vs. MLR between leukocytes and analyzing all the data reported). On the other hand, T cells of each donor of PBMC were triggered by allo-antigens but not in presence of self cells. This behavior indicates that PBMC used in these experiments were able to respond to allo-antigens. Finally, we analyzed if allogeneic hAC could inhibit MLR when added as a third party. Thus, MLR was performed on allogeneic hAC at the responder T cells:hAC ratio of 10:1 and proliferation evaluated as decrease of CFSE content in responder T cells. We have found that three different preparations of allogeneic hAC inhibited by 60–70% MLR proliferation (Figure 7B, *p* < 0.001, comparing proliferation in MLR with hAC as third part vs. proliferation of MLR and analyzing collectively the data). Altogether, these findings strongly suggest that hAC act as veto cells in allo-response.

## Discussion

Herein, we have shown that hAC can regulate immune response influencing directly the activation of T lymphocytes and indirectly impairing the differentiation of PB Mo induced by GM-CSF and IL-4 to professional APC as DC.

The inhibition of T cell proliferation was evident when hAC and lymphocyte were in close contact but not when these two cell populations were separated by a transwell system. However, soluble factors released in PBMC–hAC co-cultures passing through transwell can inhibit T cell proliferation, indicating that interaction between hAC and leukocytes is a requisite to deliver inhibiting signal. This inhibiting signal was evident on purified CD4^+^ T cells. This involved the impairment of the upregulation of IL-2R induced by appropriate stimuli interfering with IL-2/IL-2R signaling and consequent T lymphocyte proliferation. The inhibition of anti-CD3mAb-mediated proliferation would suggest that hAC can impair T cell antigen (TCR)-dependent activation (19) leading to a possible explanation of lack of allogenic stimulation of hAC observed in our experiments and reported by others (9). The impairment of proliferation with PHA or SEB support the notion that hAC can affect antigen independent T cell proliferation and triggering through bacterial products (19–21). Altogether, these observations reinforced the idea that regulatory activity of hAC on T cell response could have a role in articular joints injured by a trauma or inflamed during articular infections. hAC can strongly influence the maturation of Mo to iDC and mDC as well as their capacity to trigger as professional APC T cell proliferation. This finding support the idea that immune-regulation exerted by hAC can be exploited indirectly acting on professional APC maturation and function as well. The inhibition on Mo differentiation was evident also when very low number of hACs was co-cultured with Mo during their maturation to iDC. Indeed, just one hAC vs. 100 or more Mo can fully impaired differentiation induced through GM-CSF and IL-4; on the other hand, inhibition of T cell proliferation was detectable at 10:1 T:hAC ratio. This suggests that hAC can regulate better Mo than T cells. This finding would be relevant in the joint cartilage where Mo-derived macrophages have been reported to play a key role in rejection of hAC in a minipig model (25). Indeed, the co-localization of macrophages to hydrogel-implanted hAC suggested active graft rejection without evidence for an immune-privileged status of xenogeneic chondrocytes in this experimental system (25). In this context, it has also been reported that immune tolerance to allogeneic tissue engineered cartilage and subsequent implant survival is dependent on the implant location and proximity to the synovium (26). Furthermore, it has been shown the safety and initial efficacy of a cell-based therapy using allogeneic juvenile chondrocytes delivered percutaneously for the treatment of lumbar spondylosis with mechanical low-back pain (27). Altogether these findings would suggest that the immune response to allogeneic hAC could be dependent on the microenvironment that hAC encounter and how these hAC are delivered. This notion is further supported by the observation that stress and tissue fluid microenvironments can greatly impact the differentiation and immunological properties of the engineered cartilage (28); furthermore, the use of cultured chondrocytes in artificial scaffolds may create a temporary mechanical barrier between transplant and bone marrow during implantation concurring to reduce immunogenicity of chondrocytes (29). It has been reported that differentiated DC pulsed with supernatant from allogeneic hAC can efficiently trigger immune response and that TNF-α further increased this effect (30, 31). This suggests that already differentiated DC are insensitive to hAC-mediated regulation and that the immune response to allogeneic chondrocytes implantation can be different in relation to the presence of fully mature APC in the microenvironment.

The conditioned media (CM) from Mo–hAC co-cultures can affect the differentiation of Mo to iDC and mDC demonstrating that soluble factors can be involved. Among the different factors that could possibly play a role in immune-regulation and impairment of Mo to DC differentiation (24, 32, 33), PGE_2_ has been identified to impair Mo to DC generation. Most importantly, the presence of PGE_2_ in CM of Mo with hAC cultures suggests that PGE_2_ could be a suitable candidate as responsible factor for the impairment of Mo differentiation to DC. In this context, two relevant types of immunoregulatory cells have been reported to be sensitive to PGE_2_ increase, such as regulatory T cells (Tregs) and myeloid-derived suppressor cells (MDSC) (34–36). Thus, PGE_2_ can amplify the regulatory activity of Treg and MDSC (34–36). It is still to be defined whether in co-culture with hAC can be generated either Treg or MDSC. In this context, it is evident that intact cartilage can release several factors, such as TGF-β, that can influence the microenvironment (37) and lead to the generation of Tregs (37, 38).

Most importantly, chondrocytes can be derived from bone marrow MSC under appropriate culture conditions (39), and it has been reported that MSC-derived chondrocytes increases antidonor immune response (10). In this context, it is conceivable that some chondrocytes of articular cartilage may be derived from MSC migrated from the bone marrow. Interestingly, it has been reported (40) that MSC from bone marrow can down regulate DC maturation from peripheral Mo at a Mo:MSC ratio of 5:1 that is about 100-fold lower than that showed by hAC in our hands. This different efficiency between MSC and chondrocytes in regulating Mo differentiation can be related to the origin of the two different cell populations. Indeed, MSC were from bone marrow of young children and in that site they played a key role in the regulation of differentiation of hematopoietic cell precursors (41). In our experiments, chondrocytes are from articular cartilage of old patients and they are committed to produce factors typical of differentiated chondrocytes (Figure 1). In addition, it has been reported that MSC can regulate Mo differentiation through PGE_2_ and indoleamine dioxigenase (IDO) activity (40) while in our hands, kynurenine, a metabolite of IDO activity on tryptophan aminoacid, did not affect consistently Mo differentiation. This would suggest that some of the different experimental settings, used in our vs. previously reported experiments, could be the reason of the different inhibitory degree of chondrocytes. By the use of rat subcutaneous implantation model, it has been shown that rat chondrogenically differentiated MSC acquire a more immunogenic phenotype (10). In this experimental system, MSC differentiated into chondrocytes expressed high levels of co-stimulatory molecules as CD80 and CD86 (10). It is well known that CD80 and CD86 are accessory molecules essential for giving to T cell the second signal to trigger proliferation through the engagement of CD28 at the T cell surface (9). Although not shown, chondrocytes used in our experiments did not express CD80 and CD86 neither at the protein nor at the mRNA level. Thus, this difference is an additional putative reason, besides the origin and species, of the different behavior of chondrogenically differentiated MSC and our hAC. The premise of isolated chondrocytes immune privilege remains contested, as studies using different species and different experimental designs have supported or refused this theory. In this context, Adkisson et al. reported that juvenile chondrocytes presented a stronger immune modulatory effect than adult chondrocytes. Young chondrocytes not only failed to active T cells but also were able to inhibit activated T cells proliferation possibly by a mechanism involving the co-stimulatory molecule B7-1–CD80 (42). More recently, Lohan et al. reported that rat articular chondrocytes did not induce T cell activation and were able to modulate ongoing T cell proliferative response through the release of nitric oxide (43). Also, in the reported culture system, rat chondrocytes presented immunosuppression properties, fact that is in line with our outcomes using different approaches and different cell spices. Additionally, it was demonstrated that low doses of PGE_2_ revealed beneficial effects on the phenotypic differentiation of hAC in different culture system (44).

Despite these observations, we have to take into consideration that classic ACI requires the use of autologous chondrocytes isolated from biopsy samples. Cells expanded in monolayer inevitably undergo into dedifferentiation process, which compromise phenotypically traits. The quality of the delivered allogenic chondrocytes combined to robust effect of hAC on the proliferation and functionality of T and professional APC could play a crucial role in the inflammatory response during tissue repair process. Accordingly, allogeneic hAC do not trigger allo-immune response and they impair MLR as a third party in *in vitro* experiments, which indicates that hAC regulation of immune response can overcome allostimulation. Nevertheless, it would be useful to investigate in an appropriate animal model if chondrocytes, whether inside their dense matrix or navigating in the synovial fluid, maintain the observed immune behavior and the capacity to repair the original hyaline cartilage tissue.

## Author Contributions

RCP performed the experiments, designed the study, and wrote the paper; DM performed the experiments; RC and CG wrote the manuscript; and AP performed and designed the experiments and wrote the manuscript.

## Conflict of Interest Statement

The authors declare that the research was conducted in the absence of any commercial or financial relationships that could be construed as a potential conflict of interest.

## Supplementary Material

Figure S1 **hAC inhibit T lymphocyte proliferation**. **(A)** PBMC were isolated from peripheral blood, labeled with CFSE and co-cultured with hAC at the PBMC:hAC ratio of 5:1. PBMC were stimulated with anti-CD3 mAb (20 ng/ml) or PHA (5 μg/ml) or SEB (10 ng/ml) for 4 days and T cell proliferation was assessed as decrement of CFSE content in CD3^+^ T cells (identified by indirect immunofluorescence with anti-CD3 mAb UCHT-1 followed by goat APC-conjugated anti-mouse isotype specific antiserum). Results were shown as log green fluorescence intensity arbitrary units (a.u.) of CFSE content vs. log far red fluorescence intensity. One representative experiment out of 10 performed. Medium indicates the CFSE content and CD3 expression in unstimulated PBMC. Upper row proliferation of PBMC alone; lower row proliferation of PBMC co-cultured with hAC. **(B)** CFSE content gated on CD3^+^ T cells [corresponding to the cells depicted in upper left and upper right quadrant of **(A)**] on PBMC (upper subpanels) or PBMC co-cultured with hAC (lower subpanels) of unstimulated PBMC (medium) or PHA stimulated PBMC (right subpanels).

Figure S2 **hAC inhibit directly CD4^+^ T cell proliferation triggered through CD3 and CD28**. **(A)** Highly purified peripheral blood CD4^+^ T cells labeled with CFSE were co-cultured with hAC at the CD4^+^ T cell:hAC ratio of 5:1 in the presence of anti-CD3 and anti-CD28 coated beads and proliferation was assessed on day 3 by FACS analysis of CFSE decrement compared to unstimulated CD4^+^ T lymphocytes (medium). Lower subpanels show the proliferation on day 3 of CD4^+^ T cells in the presence of exogenous IL-2 added at day 2 of stimulation assay. Results are representative of 10 different experiments using 6 different preparations of hAC and 10 donors of CD4^+^ T cells. **(B)** Images of cell cultures of **(A)**.

Figure S3 **Features of peripheral Mo cultured with hAC**. Mo were isolated from PBMC as CD14^+^ cells by Easy-Sep do-it yourself kit and analyzed for the expression of CD14 and CD16 to identify different Mo subsets by indirect immunofluorescence and FACS analysis (45). **(A)** left. Physical parameters as forward scatter (FSC, *x*-axis) and side scatter (SSC, *y*-axis) of total peripheral blood mononuclear cells (PBMC), three different populations can be identified: low FSC–low SSC: debris-platelets; high FSC–low SSC: lymphocytes; and high FSC–high SSC Mo (circled by gate). **(A)**, right. FSC and SSC of CD14^+^ purified Mo; **(B)** Double immunofluorescence of purified CD14^+^ cells from PBMC with anti-CD14 mAb (*x*-axis) and anti-CD16 mAb (*y*-axis) followed by anti-isotype-specific PE and Alexafluor 647 goat anti-mouse antiserum. **(C)** FSC and SSC of Mo cultured for 6 days with GM-CSF (20 ng/ml) and IL-4 (20 ng/ml) without (upper dot plot) or with hAC (lower dot plot). Circled dots were those analyzed for the expression of the different differentiation markers shown in Figure 4. Lymphocytes represented less than 5% of total cell population used for the analysis. **(D)** Morphological features of Mo cultured for 6 days without (left) or with hAC (right) in medium (upper images) or with GM-CSF + IL-4 (lower images). Images were taken with IX70 Olympus inverted microscope at 100× magnification equipped with an ORCA Hamamatsu camera. Bar: 10 μm.

Figure S4 **Effect of hAC on peripheral blood Mo to dendritic cell differentiation**. Immunofluorescence analysis of Mo isolated from peripheral blood (PB monocytes) (first upper row), or cultured with GM-CSF (20 ng/ml) and IL-4 (20 ng/ml) (second row) or with GM-CSF + IL-4 and hAC (third row) or with GM-CSF + IL-4 + LPS (1 μg/ml) (fourth row) or with GM-CSF + IL-4 + LPS and hAC (fifth row). GM-CSF and IL-4 (6 days of culture) were used to induce differentiation of PB Mo to iDC (second and third rows), while the addition of LPS (6 days of culture + additional 2 days with LPS) was used to further induce differentiation of iDC to mDC (fourth and fifth rows). Cells were harvested from cell culture wells and labeled with anti-CD1a-FITC, anti-CD14-PE-Cy7, anti-CD80-Violet, anti-CD83-APC, and anti-CD86-PE-conjugated antibodies. Five-color immunofluorescence analysis was performed with the CyAN ADP cytofluorimeter and at least 10,000 events for each dot plot have been analyzed. Analysis was performed on gated Mo as indicated in Figure S2 in Supplementary Material. Each subpanel was divided into four quadrants (upper left, single-positive cells for the indicated markers on *y*-axis; upper right double-positive cells co-expressing CD1a; lower left double-negative cells; lower right single CD1a^+^ cells) on the basis of negative controls stained with an unrelated mAb matched for the isotype. Optimal compensation between the various color was performed using auto-comp included in Summit analysis program and confirmed by the staining of cells with one or another single antibody. Results are expressed as mean Log green fluorescence intensity of CD1a and the mean of fluorescence intensity of each marker indicated on *y*-axis in arbitrary units (a.u.). Results are 1 out of 15 independent experiments using different hAC preparations and 8 Mo donors.

Figure S5 **Effect of conditioned media from Mo–hAC co-cultures on Mo differentiation to iDC**. Mo or hAC or Mo and hAC were cultured with GM-CSF and IL-4 for 4 days. Then, conditioned media were harvested and added (1 in 2 diluted) to Mo induced to differentiate to iDC with GM-CSF and IL-4. On day 3 (upper dot plots) and day 6 (lower dot plots), cells were harvested and stained with anti-CD1a-FITC and anti-CD14PE-Cy7 mAbs. Samples were run on a CyAN ADP cytofluorimeter and results are representative of three independent experiments.
