# Supplementary figures and images for: Human Articular Chondrocytes Regulate Immune Response by Affecting Directly T Cell Proliferation and Indirectly Inhibiting Monocyte Differentiation to Professional Antigen-Presenting Cells
Source: Front Immunol. 2016 Oct 24;7:415. doi: 10.3389/fimmu.2016.00415 (PMC5075572; doi:10.3389/fimmu.2016.00415)

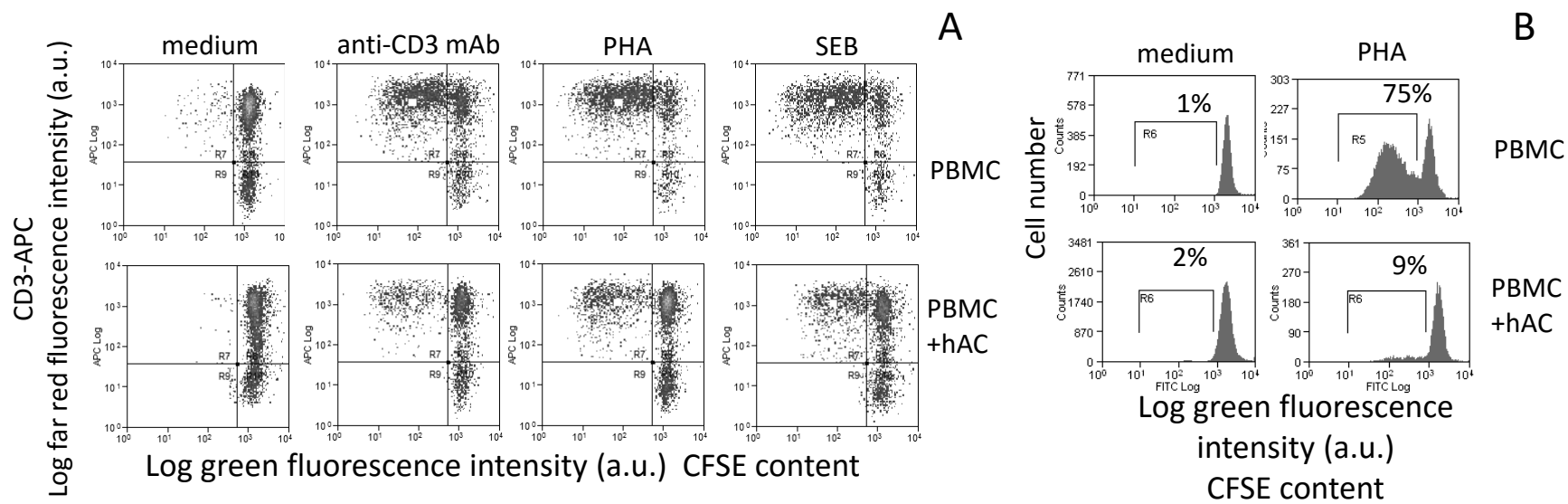

Supplemental figure 1

Supplement: Figure S1 — hAC inhibit T lymphocyte proliferation. (A) PBMC were isolated from peripheral blood, labeled with CFSE and co-cultured with hAC at the PBMC:hAC ratio of 5:1. PBMC were stimulated with anti-CD3 mAb (20 ng/ml) or PHA (5 μg/ml) or SEB (10 ng/ml) for 4 days and T cell proliferation was assessed as decrement of CFSE content in CD3+ T cells (identified by indirect immunofluorescence with anti-CD3 mAb UCHT-1 followed by goat APC-conjugated anti-mouse isotype specific antiserum). Results were shown as log green fluorescence intensity arbitrary units (a.u.) of CFSE content vs. log far red fluorescence intensity. One representative experiment out of 10 performed. Medium indicates the CFSE content and CD3 expression in unstimulated PBMC. Upper row proliferation of PBMC alone; lower row proliferation of PBMC co-cultured with hAC. (B) CFSE content gated on CD3+ T cells [corresponding to the cells depicted in upper left and upper right quadrant of (A)] on PBMC (upper subpanels) or PBMC co-cultured with hAC (lower subpanels) of unstimulated PBMC (medium) or PHA stimulated PBMC (right subpanels). [file Image_1.PDF]

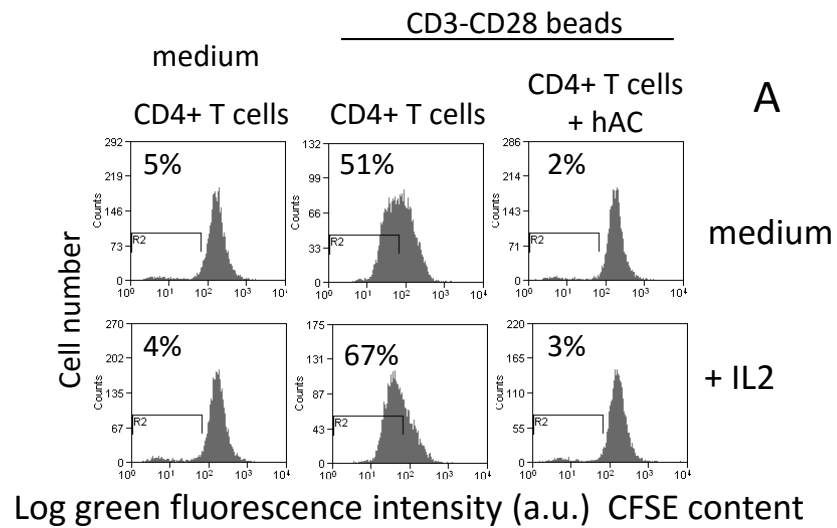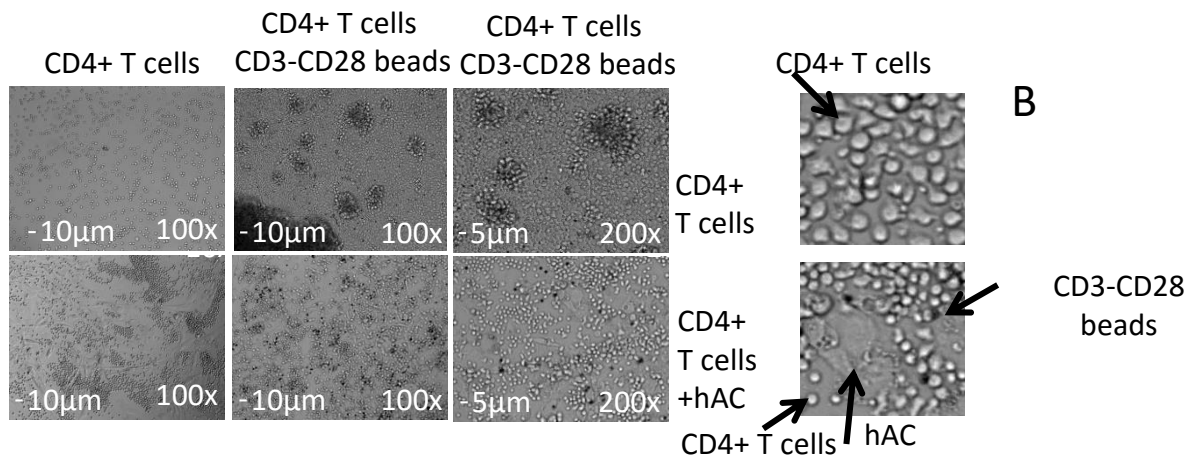

Supplement: Figure S2 — hAC inhibit directly CD4+ T cell proliferation triggered through CD3 and CD28. (A) Highly purified peripheral blood CD4+ T cells labeled with CFSE were co-cultured with hAC at the CD4+ T cell:hAC ratio of 5:1 in the presence of anti-CD3 and anti-CD28 coated beads and proliferation was assessed on day 3 by FACS analysis of CFSE decrement compared to unstimulated CD4+ T lymphocytes (medium). Lower subpanels show the proliferation on day 3 of CD4+ T cells in the presence of exogenous IL-2 added at day 2 of stimulation assay. Results are representative of 10 different experiments using 6 different preparations of hAC and 10 donors of CD4+ T cells. (B) Images of cell cultures of (A). [file Image_2.PDF]

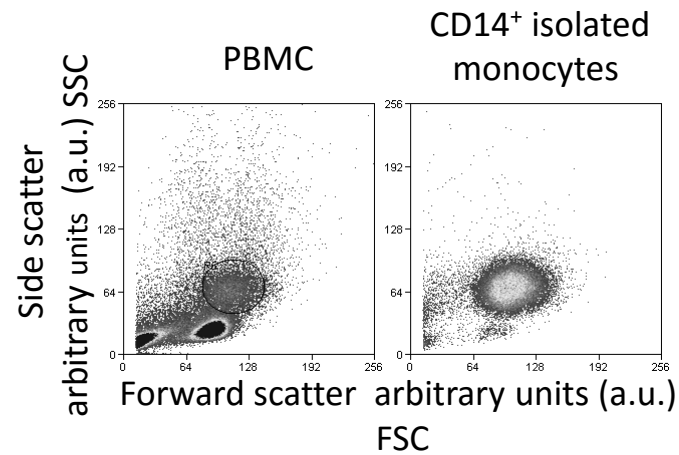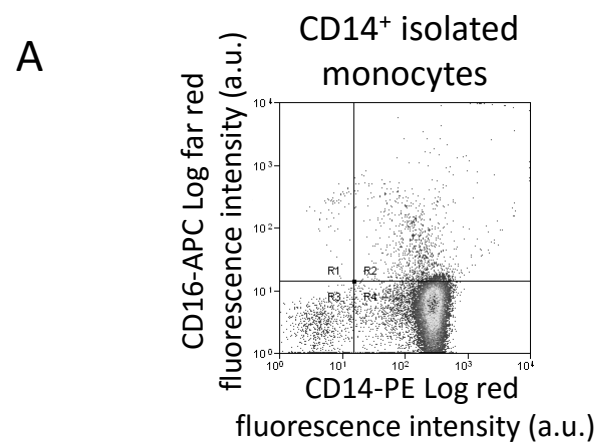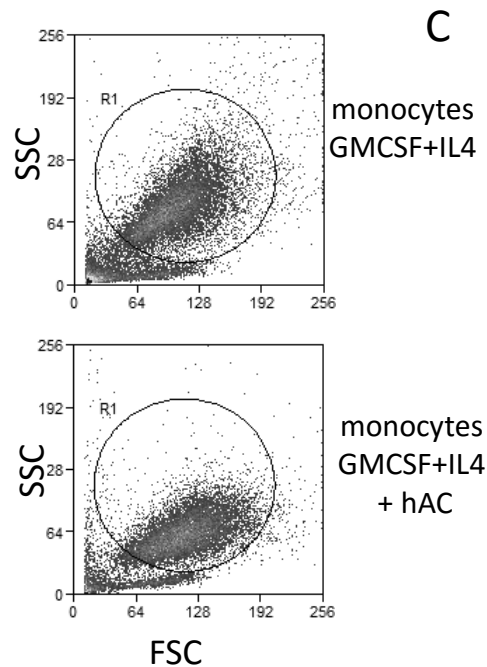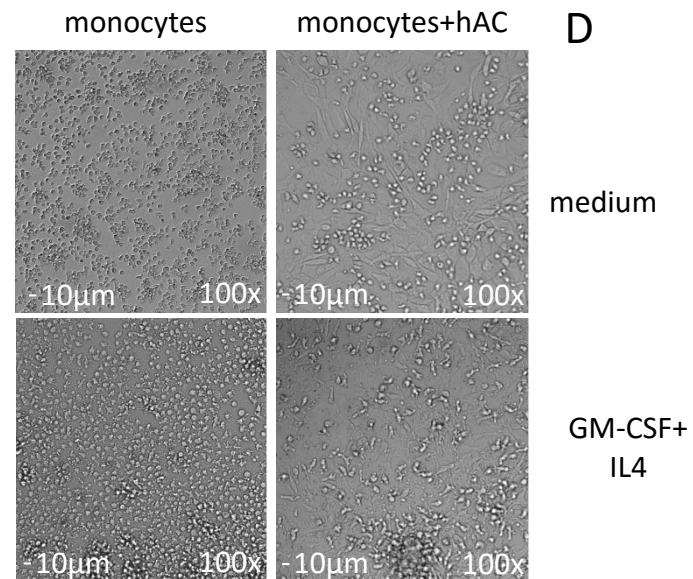

Supplemental figure 3

Supplement: Figure S3 — Features of peripheral Mo cultured with hAC. Mo were isolated from PBMC as CD14+ cells by Easy-Sep do-it yourself kit and analyzed for the expression of CD14 and CD16 to identify different Mo subsets by indirect immunofluorescence and FACS analysis (45). (A) left. Physical parameters as forward scatter (FSC, x-axis) and side scatter (SSC, y-axis) of total peripheral blood mononuclear cells (PBMC), three different populations can be identified: low FSC–low SSC: debris-platelets; high FSC–low SSC: lymphocytes; and high FSC–high SSC Mo (circled by gate). (A), right. FSC and SSC of CD14+ purified Mo; (B) Double immunofluorescence of purified CD14+ cells from PBMC with anti-CD14 mAb (x-axis) and anti-CD16 mAb (y-axis) followed by anti-isotype-specific PE and Alexafluor 647 goat anti-mouse antiserum. (C) FSC and SSC of Mo cultured for 6 days with GM-CSF (20 ng/ml) and IL-4 (20 ng/ml) without (upper dot plot) or with hAC (lower dot plot). Circled dots were those analyzed for the expression of the different differentiation markers shown in Figure 4. Lymphocytes represented less than 5% of total cell population used for the analysis. (D) Morphological features of Mo cultured for 6 days without (left) or with hAC (right) in medium (upper images) or with GM-CSF + IL-4 (lower images). Images were taken with IX70 Olympus inverted microscope at 100× magnification equipped with an ORCA Hamamatsu camera. Bar: 10 μm. [file Image_3.PDF]

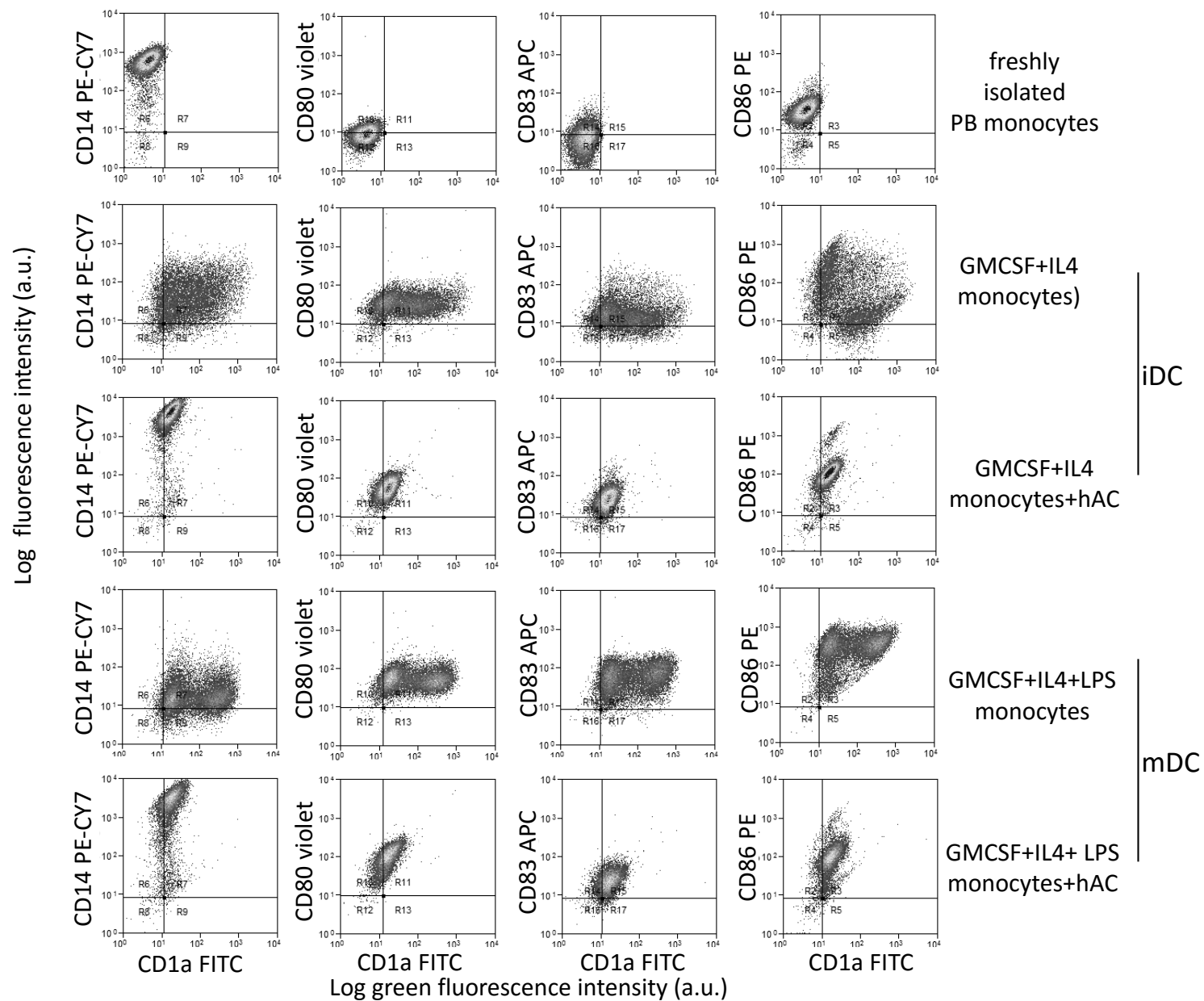

Supplemental figure 4

Supplement: Figure S4 — Effect of hAC on peripheral blood Mo to dendritic cell differentiation. Immunofluorescence analysis of Mo isolated from peripheral blood (PB monocytes) (first upper row), or cultured with GM-CSF (20 ng/ml) and IL-4 (20 ng/ml) (second row) or with GM-CSF + IL-4 and hAC (third row) or with GM-CSF + IL-4 + LPS (1 μg/ml) (fourth row) or with GM-CSF + IL-4 + LPS and hAC (fifth row). GM-CSF and IL-4 (6 days of culture) were used to induce differentiation of PB Mo to iDC (second and third rows), while the addition of LPS (6 days of culture + additional 2 days with LPS) was used to further induce differentiation of iDC to mDC (fourth and fifth rows). Cells were harvested from cell culture wells and labeled with anti-CD1a-FITC, anti-CD14-PE-Cy7, anti-CD80-Violet, anti-CD83-APC, and anti-CD86-PE-conjugated antibodies. Five-color immunofluorescence analysis was performed with the CyAN ADP cytofluorimeter and at least 10,000 events for each dot plot have been analyzed. Analysis was performed on gated Mo as indicated in Figure S2 in Supplementary Material. Each subpanel was divided into four quadrants (upper left, single-positive cells for the indicated markers on y-axis; upper right double-positive cells co-expressing CD1a; lower left double-negative cells; lower right single CD1a+ cells) on the basis of negative controls stained with an unrelated mAb matched for the isotype. Optimal compensation between the various color was performed using auto-comp included in Summit analysis program and confirmed by the staining of cells with one or another single antibody. Results are expressed as mean Log green fluorescence intensity of CD1a and the mean of fluorescence intensity of each marker indicated on y-axis in arbitrary units (a.u.). Results are 1 out of 15 independent experiments using different hAC preparations and 8 Mo donors. [file Image_4.PDF]

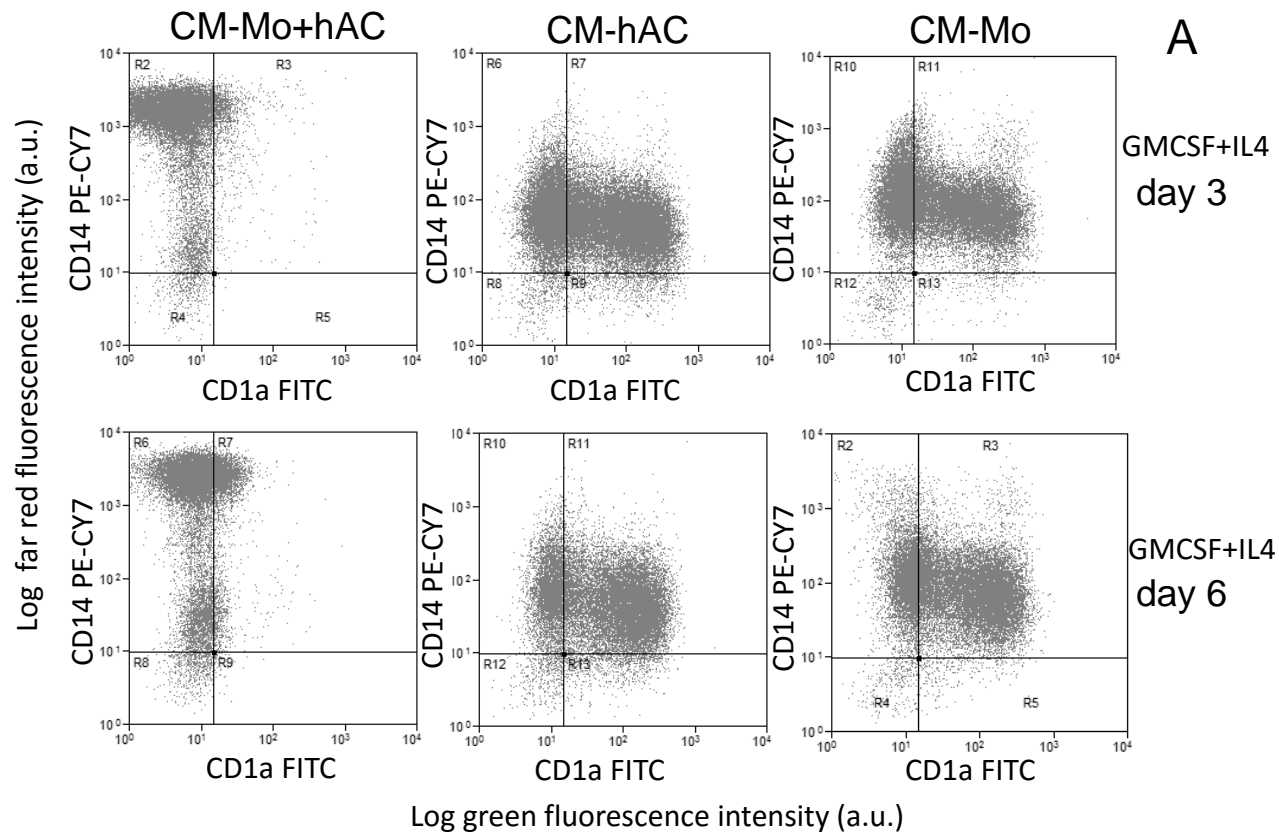

Supplement: Figure S5 — Effect of conditioned media from Mo–hAC co-cultures on Mo differentiation to iDC. Mo or hAC or Mo and hAC were cultured with GM-CSF and IL-4 for 4 days. Then, conditioned media were harvested and added (1 in 2 diluted) to Mo induced to differentiate to iDC with GM-CSF and IL-4. On day 3 (upper dot plots) and day 6 (lower dot plots), cells were harvested and stained with anti-CD1a-FITC and anti-CD14PE-Cy7 mAbs. Samples were run on a CyAN ADP cytofluorimeter and results are representative of three independent experiments. [file Image_5.PDF]
